# The Role of Self-Esteem in Parent–Child Conflict and Problematic Smartphone Use Among Adolescents: A Three-Wave Cross-Lagged Study

**DOI:** 10.3390/bs16101881

**Published:** 2026-10-12

**Authors:** Shuang Lin, Ziyi Luo, Yongjian Li, Jing Sheng, Lijun Chen, Huifang Yang, Jiamin Liang, Jun Chen

**Affiliations:** 1 School of Educational Science, Guangdong Polytechnic Normal University, Guangzhou 510665, China; linshuang@gpnu.edu.cn (S.L.);; 2 School of Psychology, South China Normal University, 55 West Zhongshan Road, Guangzhou 510631, China; 3 Key Laboratory of Psychology of TCM and Brain Science, Jiangxi Administration of Traditional Chinese Medicine, Jiangxi University of Chinese Medicine, Nanchang 330006, China

**Keywords:** parent–child conflict, problematic smartphone use, self-esteem, Person–Affect–Cognition–Execution model

## Abstract

Although prior research has linked parent–child conflict to problematic smartphone use (PSU), most evidence is cross-sectional, leaving the directionality unclear. Guided by the Interaction of Person–Affect–Cognition–Execution (I-PACE) model, this three-wave longitudinal study examined the reciprocal links between parent–child conflict and PSU and the mediating role of self-esteem. Chinese adolescents (N = 1368; 40% female; Mage = 16.05, SD = 0.85 at baseline) completed self-report measures of parent–child conflict, self-esteem, and PSU at six-month intervals. Cross-lagged panel models (CLPMs) were the primary analysis, and random-intercept CLPMs (RI-CLPMs) served as a sensitivity analysis. CLPMs showed bidirectional predictions between parent–child conflict and PSU, with T2 self-esteem mediating both indirect paths. RI-CLPMs revealed significant between-person associations among parent–child conflict, self-esteem, and PSU; at the within-person level, only T1 parent–child conflict prospectively predicted T2 PSU, whereas the reverse path and within-person mediation were not significant. These findings suggested that the longitudinal link between parent–child conflict and PSU may partly reflect stable between-person differences, with only limited within-person prospective evidence for a conflict-to-PSU pathway.

## 1. Introduction

Smartphones have spread rapidly across the globe due to their powerful functions and convenience. In 2023, the internet penetration rate among minors in China reached 97.2%, with 90% of minors primarily using smartphones to access the internet (CNNIC, 2023). Alongside benefits, PSU among adolescents has become increasingly evident. A meta-analysis of 41 studies (41,871 children and adolescents) found a median PSU prevalence of 23.3% (Sohn et al., 2019). PSU refers to excessive or maladaptive smartphone use that arises from an individual’s inability to regulate smartphone usage, leading to problems at psychological, physiological, and behavioral levels, with potential addictive properties (Billieux et al., 2015; Panova & Carbonell, 2018). Although the manifestations of PSU resemble those of addiction and include features such as loss of control and help-seeking (Tao et al., 2017), the three core characteristics of addiction (loss of control, tolerance, and withdrawal) have received limited empirical support in the context of smartphone use. It is not recognized as a clinical addiction in DSM-5; we therefore treat it as a behavior with potential addictive properties. PSU impairs adolescents’ sleep, mental health, and social functioning (Fischer-Grote et al., 2021; Y. Liu et al., 2025; Zhang et al., 2022), making it essential to identify its mechanisms.

Parent–child conflict has been consistently associated with PSU (Wei et al., 2025; Zhu et al., 2022). However, whether this association exists is no longer novel. The key questions are how, in which direction, and through which mechanism it unfolds. Most studies use cross-sectional, unidirectional designs and cannot determine whether conflict precedes PSU, PSU elicits conflict, or both reinforce each other. Longitudinal cross-lagged panel models (CLPMs) are needed to establish temporal precedence and test reciprocity. This study therefore examines the longitudinal reciprocal relationship between parent–child conflict and PSU and tests self-esteem as a mediator in both directions.

### 1.1. The Relationship Between Parent–Child Conflict and Problematic Smartphone Use

Parent–child relationships are central to adolescent development. Parent–child conflict refers to discrepancies or oppositions between adolescents and parents in perspectives, expectations, and behaviors, involving negative emotional experiences and overt interaction (Bradford et al., 2008; Fang & Dong, 1998). Family systems theory views the family as an interdependent emotional system in which imbalanced interactions may produce persistent maladjustment (Bowen, 1966). Conflict disrupts stable emotional exchange; lacking support and communication, adolescents may withdraw and use smartphones to escape tense family atmospheres (Deng et al., 2013; Wei et al., 2025). Parent–child conflict is thus a risk factor for PSU (Ko et al., 2015; Özaslan et al., 2022).

Family systems theory also holds that family interactions are bidirectional; children’s behavior shapes parents’ attitudes and behaviors (Bowen, 1966). PSU may therefore trigger negative parental responses and worsen conflict. Although prospective evidence is scarce, indirect evidence supports this pathway. Compulsive internet use predicts poorer parent–child communication and lower parental care over time (Van den Eijnden et al., 2010). Excessive smartphone use may deplete emotional resources needed for stress coping (Brand et al., 2019), making adolescents irritable and reactive in face-to-face interactions, misreading parental concern as intrusion (Peng et al., 2025; Zhou et al., 2024) and increasing conflict frequency and intensity (Q. Q. Liu et al., 2018). Thus, higher PSU may predict greater later parent–child conflict. Accordingly:

**Hypothesis** **1.** *There is a longitudinal reciprocal relationship between parent–child conflict and PSU. Specifically, frequent parent–child conflict may predict increased PSU; conversely, higher levels of PSU may predict greater subsequent parent–child conflict.*

### 1.2. The Mediating Role of Self-Esteem

Family factors are closely tied to adolescents’ self-evaluation, and self-esteem may mediate the bidirectional relationship between parent–child conflict and PSU. Self-esteem refers to satisfaction with oneself based on self-worth evaluation, capturing positive and negative attitudes toward the self (Brailovskaia & Margraf, 2020; Crocker & Wolfe, 2001). The I-PACE model (Brand et al., 2016) posits that problematic Internet use emerges from interactions among Person, Affect, Cognition, and Execution; repeated cycles may impair executive control and create a self-perpetuating stimulus–affect–cognition–behavior cycle. Although PSU is not a clinical addiction, it shares features with behavioral addictions, making I-PACE useful. Parent–child conflict acts as a persistent stressor that undermines self-esteem (Jeong et al., 2020). Low self-esteem is a vulnerability factor for compensatory smartphone expectations and negative emotions (Kim, 2022; Csibi et al., 2021), impairing executive control and leading to loss of control over smartphone use (Brand et al., 2019). Immediate gratification may reinforce maladaptive schemas and excessive use (Csibi et al., 2021); negative consequences such as sleep deprivation and social isolation may further worsen emotions and intensify the cycle (Casale et al., 2022; Zhang et al., 2022).

On the one hand, parent–child conflict may indirectly predict PSU by eroding self-esteem. Chronic conflict exposes adolescents to negative feedback, reducing self-worth (Jia et al., 2024). Low self-esteem increases desire for escape and external recognition (Brand et al., 2019), and reliance on smartphones to rebuild identity and gain affirmation (Kim, 2022; Kong et al., 2020; Peng et al., 2025; Zhou et al., 2024; Özaslan et al., 2022). Evidence shows family tension is associated with low self-esteem (Jeong et al., 2020; Kim, 2022; Tian et al., 2018). Moreover, cross-developmental evidence from preadolescent samples further confirms that perceived parental rejection and hostility, as typical forms of negative family interaction, are associated with impaired self-perception (Christou et al., 2026), lending convergent support to the association between negative family relational experiences and individuals’ self-evaluation. In addition, adolescents with both severe parent–child conflict and low self-esteem show a higher risk of PSU (Jeong et al., 2020; A. Wang et al., 2024). On the other hand, PSU may induce parent–child conflict via self-esteem. Within the I-PACE model, PSU involves a loss of control over smartphone use. Loss of control during digital engagement and subsequent post-use regret may fuel self-criticism and erode adolescents’ self-esteem (Snegha & Sudha, 2025), consistent with evidence documenting negative correlations between severe PSU and diminished self-perception (Casale et al., 2022; Mathew & Krishnan, 2020). Fragile self-esteem may lead adolescents to externalize negative emotions and interpret parental reminders as criticism or hostility, escalating disagreements (Snegha & Sudha, 2025; Orth & Robins, 2013; Zhou et al., 2024). Parental corrective pressure may further aggravate tension (Brand et al., 2019; Csibi et al., 2021; Efrati et al., 2024). Thus, self-esteem may mediate both pathways.

Using the I-PACE model as the framework and integrating the evidence reviewed above, the expected reciprocal pathways between parent–child conflict and PSU may be indirectly driven by self-esteem. To clarify the longitudinal predictive linkage between parent–child conflict and PSU as well as the mediating role of self-esteem, the following hypothesis is proposed:

**Hypothesis** **2.** *Self-esteem would mediate the indirect pathways between parent–child conflict and PSU in both directions. Specifically, higher parent–child conflict was expected to predict lower self-esteem, which in turn would predict higher PSU; conversely, higher PSU was expected to predict lower self-esteem, which in turn would predict higher parent–child conflict.*

### 1.3. The Current Study

Guided by the I-PACE model, this study used CLPMs with 1368 Chinese adolescents to investigate (a) whether a reciprocal relationship exists between parent–child conflict and PSU, and (b) whether self-esteem mediates this reciprocal relationship. Demographic covariates, including age and gender, were controlled for in all analyses.

## 2. Methods

### 2.1. Participants

The present study utilized data from a larger longitudinal project on adolescent mental health (Lin et al., 2025), with participants recruited from four schools in Guangdong Province, China. We adopted a short-term longitudinal design with three data collection waves spaced at six-month intervals across one year. A total of 1655 adolescents completed the baseline assessment (T1); 1498 participated at T2 (9.5% attrition relative to T1); and 1368 participated at T3 (17.3% total attrition relative to T1; 8.7% attrition from T2 to T3). The final analytical sample therefore comprised 1368 adolescents (60.0% male; mean age at baseline = 16.05 ± 0.85 years). The primary reason for attrition was students leaving school or being absent on the day of the survey. We compared the retained sample (n = 1368) with the attrited participants (n = 287) on all key study variables, including parent–child conflict, PSU, self-esteem, gender, age, parental education, and family socioeconomic status. The results showed that the two groups did not differ significantly in gender, χ^2^(1) = 3.14, *p* = 0.076, Cramér’s V = 0.044, parental education (father: *t* = 1.59, *p* = 0.111; mother: *t* = 1.50, *p* = 0.133), family socioeconomic status (*t* = 0.10, *p* = 0.924), T1 parent–child conflict (*t* = 0.40, *p* = 0.687), T1 self-esteem (*t* = 0.34, *p* = 0.734), or T1 PSU (*t* = 0.19, *p* = 0.851). However, attrited participants were slightly older than retained participants (*t* = −4.28, *p* < 0.001).

The final retained cohort consisted of 1368 adolescents. Regarding family structure, 78.9% of the participants grew up with siblings, whereas 21.1% were single children. An examination of parental education levels indicated that the majority of fathers (66.2%) and mothers (71.7%) had completed high school or below. Furthermore, associate degrees were held by 24.6% of fathers and 20.5% of mothers, bachelor’s degrees by 6.3% and 5.4%, and postgraduate qualifications by 3.0% and 2.4%, respectively. In terms of the wealth status of the participants’ families, 82.0% perceived their socioeconomic standing as middle-income, with the remaining participants categorizing their households as extremely wealthy (0.4%), comfortably off (4.2%), somewhat impoverished (10.5%), or severely poor (2.9%).

### 2.2. Measures

#### 2.2.1. Parent–Child Conflict

Parent–child conflict was measured with the 8-item Chinese adaptation of the Parent–Child Conflict Questionnaire (Fang & Dong, 1998). Participants retrospectively reported the frequency of conflicts with their parents in the prior six months (e.g., I frequently argue with my parents about my academic performance), responding to all items on a 5-point Likert scale (1 = “*never*”, 5 = “*several times a day*”). Higher mean scores indicated greater frequency of parent–child conflict. The scale has demonstrated good reliability and validity among Chinese adolescents (Liang et al., 2024). The Chinese version of the scale has been validated among Chinese adolescents (Hu et al., 2019), which supported good construct validity: χ^2^/df = 6.46, CFI = 0.96, TLI = 0.94, RMSEA = 0.04. In the current study, the Cronbach’s alpha coefficients were 0.89, 0.90, and 0.91 at T1, T2, and T3, respectively.

#### 2.2.2. Problematic Smartphone Use

PSU was measured using the Chinese version of the Mobile Phone Addiction Index (MPAI; Leung, 2008), which consists of 17 items across four dimensions: loss of control (e.g., I spend much more time on my mobile phone than I originally planned.), withdrawal (e.g., I feel anxious and distracted if I cannot check my phone messages for several hours.), escape (e.g., I pick up my mobile phone to relieve stress or escape from my troubles.), and inefficiency (e.g., Excessive mobile phone use delays my homework and daily responsibilities.). All items are rated on a 5-point scale (1 = “*never*”, 5 = “*always*”), with higher mean scores indicating higher levels of PSU. The scale has demonstrated good reliability and validity among Chinese adolescents. The Chinese version has been validated among Chinese adolescents (Jiang et al., 2022). CFA showed good fit: χ^2^/df = 5.13, NFI = 0.91, CFI = 0.93, GFI = 0.93, RMSEA = 0.06. In the current study, the Cronbach’s alpha coefficients were 0.93, 0.95, and 0.96 at T1, T2, and T3, respectively.

#### 2.2.3. Self-Esteem

Self-esteem was measured using the Rosenberg Self-Esteem Scale (SES; Rosenberg et al., 1995), which consists of 10 items (e.g., I feel that I am a person of equal worth to others) and uses a 4-point Likert scale (1–4 points), with higher scores indicating higher levels of self-esteem. The Chinese version has been validated in Chinese adolescent samples (Guo, 2023). CFA indicated acceptable model fit: χ^2^/df = 5.34, GFI = 0.97, CFI = 0.95, TLI = 0.93, RMSEA = 0.07, SRMR = 0.05. In the current study, the Cronbach’s alpha coefficients were 0.85, 0.89, and 0.90 at T1, T2, and T3, respectively.

### 2.3. Procedure

The author’s university ethics committee granted approval for this research. Informed consent was obtained from participating adolescents, their guardians, and the respective schools. Data were collected via questionnaires administered during regular class sessions, with completion requiring approximately 25 min. Before data collection, students were informed about the principles of confidentiality and voluntary participation, and that they could withdraw at any time without penalty. After completing the questionnaires, students received a small token of appreciation.

### 2.4. Data Analysis

First, descriptive and correlation analyses were conducted in SPSS 25.0. CLPMs were estimated in Mplus 8.0 using maximum likelihood (ML). Item-level missing data were handled with full information maximum likelihood (FIML); wave-level attrition was not imputed, and the final analytic sample comprised respondents who completed all three waves. Standard errors and 95% confidence intervals were obtained using 5000 bootstrap resamples. Model fit was evaluated using χ^2^/df, CFI, TLI, RMSEA, and SRMR; acceptable fit was defined as χ^2^/df ≤ 5, CFI ≥ 0.90, TLI ≥ 0.90, RMSEA ≤ 0.08, and SRMR ≤ 0.08 (Kline, 2005).

We first estimated an unconstrained CLPM with all autoregressive and cross-lagged paths freely estimated, and then a constrained CLPM with corresponding cross-lagged paths constrained to be equal across waves while autoregressive paths remained freely estimated. The two models were compared using a chi-square difference test; the constrained model was retained only if constraints did not significantly worsen fit. To test bidirectionality between parent–child conflict and PSU, four competing models were compared, with autoregressive paths freely estimated and cross-lagged paths constrained equal across waves: (1) full reciprocal model (conflict → PSU and PSU → conflict); (2) conflict → PSU only; (3) PSU → conflict only; and (4) no cross-lagged paths. Model differences were tested with likelihood ratio tests (∆χ^2^), and the final model was selected based on fit indices.

Next, self-esteem was added to the retained CLPM to test two indirect pathways: parent–child conflict → self-esteem → PSU and PSU → self-esteem → parent–child conflict. Indirect effects were tested with 5000 bootstrap resamples; a 95% confidence interval not containing zero indicated a significant mediation effect. Adolescent age and gender (1 = female, 0 = male) were included as covariates in all cross-lagged structural equation models. Sensitivity analyses additionally included father’s education, mother’s education, and family SES as covariates predicting T2 and T3 parent–child conflict and PSU, with T1 conflict and PSU allowed to correlate with these covariates.

To examine school-level clustering, the main model was re-estimated with cluster-robust standard errors using school as the cluster variable. Because only four schools were involved, this analysis was treated as a sensitivity check rather than a definitive adjustment. Finally, to separate stable between-person differences from within-person changes, RI-CLPMs were estimated as sensitivity analyses: a two-variable RI-CLPM for parent–child conflict and PSU, and a three-variable RI-CLPM including self-esteem as a within-person mediator. Random intercepts were specified for repeatedly measured variables, and wave-specific within-person latent variables were estimated with autoregressive and cross-lagged paths. RI-CLPMs were estimated with ML and 5000 bootstrap resamples, and fit was evaluated using χ^2^, RMSEA, CFI, TLI, and SRMR.

## 3. Results

### 3.1. Descriptive Analyses

Descriptive correlations, means, and standard deviations among all observed variables are presented in Table 1. Consistent with expectations, significant correlations exist between parent–child conflict, self-esteem, and PSU.

### 3.2. Cross-Lagged Association Between Parent–Child Conflict and Problematic Smartphone Use

**Figure 1:**
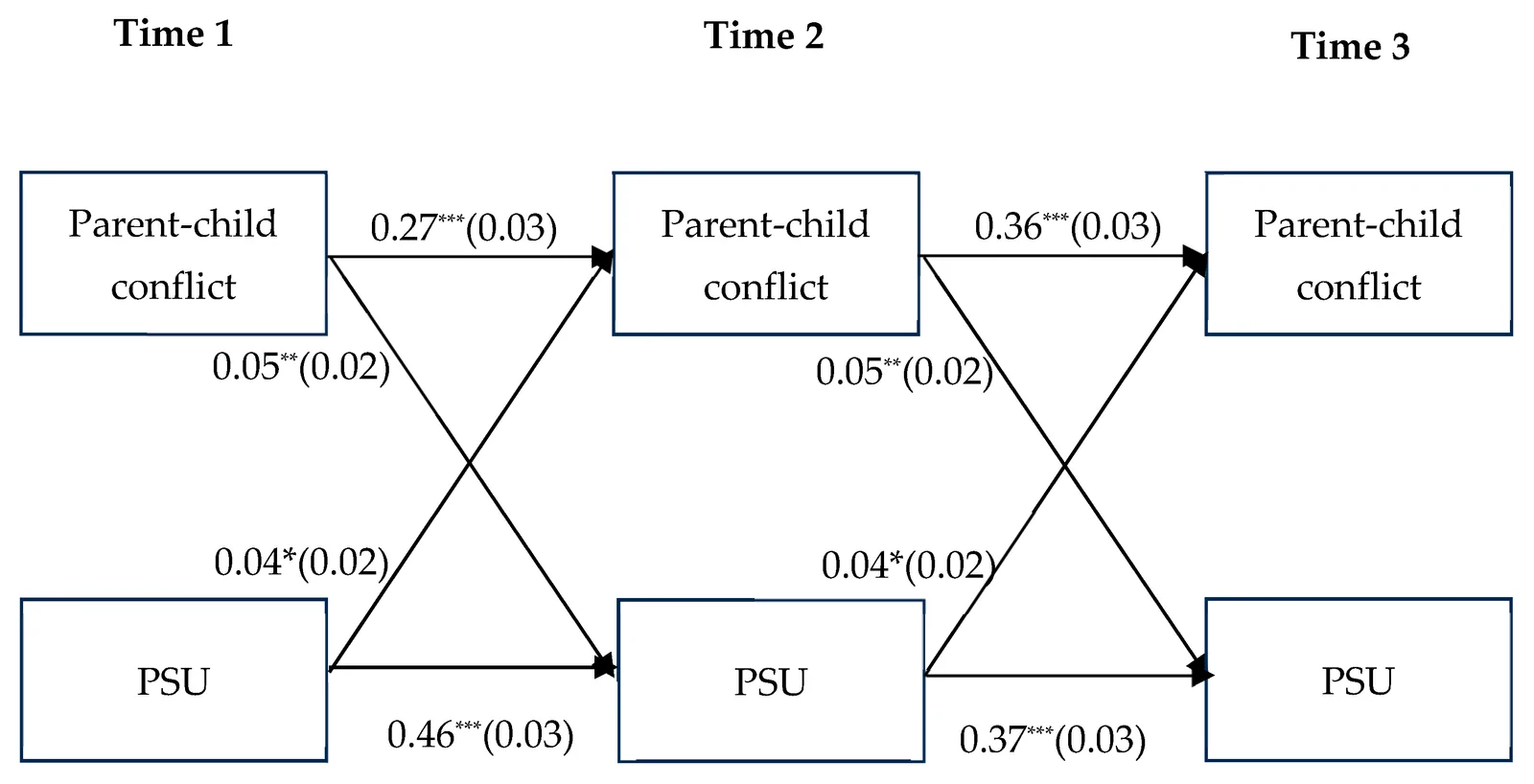
The path coefficients for the CLPM for parent–child conflict and PSU. *Note.* The values outside the parentheses are the standardized path coefficients, and the values inside the parentheses are the standard errors. Covariates, within-time correlations and second-order autoregressive paths have been estimated but are not shown in the figure to reduce complexity (* *p* < 0.05, ** *p* < 0.01, *** *p* < 0.001).

The unconstrained CLPM fitted the data well, χ^2^(2) = 2.709, χ^2^/df =1.355, RMSEA = 0.016, CFI = 0.999, TLI = 0.994, SRMR = 0.005. The constrained model, in which the corresponding cross-lagged paths were constrained to equality across waves, also fitted the data well, χ^2^(4) = 7.433, χ^2^/df = 1.858, RMSEA = 0.025, CFI = 0.997, TLI = 0.985, SRMR = 0.009. The chi-square difference test indicated that the equality constraints did not significantly worsen model fit, Δχ^2^(2) = 4.724, *p* = 0.094. Therefore, the constrained model was retained as the final, more parsimonious model. Model comparison results across four nested structural equation models are presented in Table 2. Chi-square difference tests revealed that Model 1 exhibited significantly better data fit than the three constrained alternative models (Models 2–4), identifying Model 1 as the optimal specification. Model 1 demonstrated satisfactory model fit indices: *χ*^2^(4) = 7.433, *χ*^2^/df = 1.858, RMSEA = 0.025, CFI = 0.997, TLI = 0.985, SRMR = 0.009. Examination of cross-lagged paths within Model 1 indicated that parent–child conflict significantly positively predicted subsequent PSU (*β* = 0.05, *SE* = 0.02, *p* < 0.01). The reverse cross-lagged path from PSU to later parent–child conflict was also significant (*β* = 0.04, *SE* = 0.02, *p* < 0.05). The full standardized structural path estimates are illustrated in Figure 1.

We also conducted a clustering analysis at the school level. When standard errors were adjusted for school-level clustering, the point estimates were consistent with the main model. The cross-lagged paths remained in the same direction and were of similar magnitude. However, due to the small number of clusters (four schools), the cluster-robust standard errors may not be reliable, and the significance tests should be interpreted with caution.

### 3.3. Longitudinal Indirect Effect of Self-Esteem on the Association Between Parent–Child Conflict and Problematic Smartphone Use

**Figure 2:**
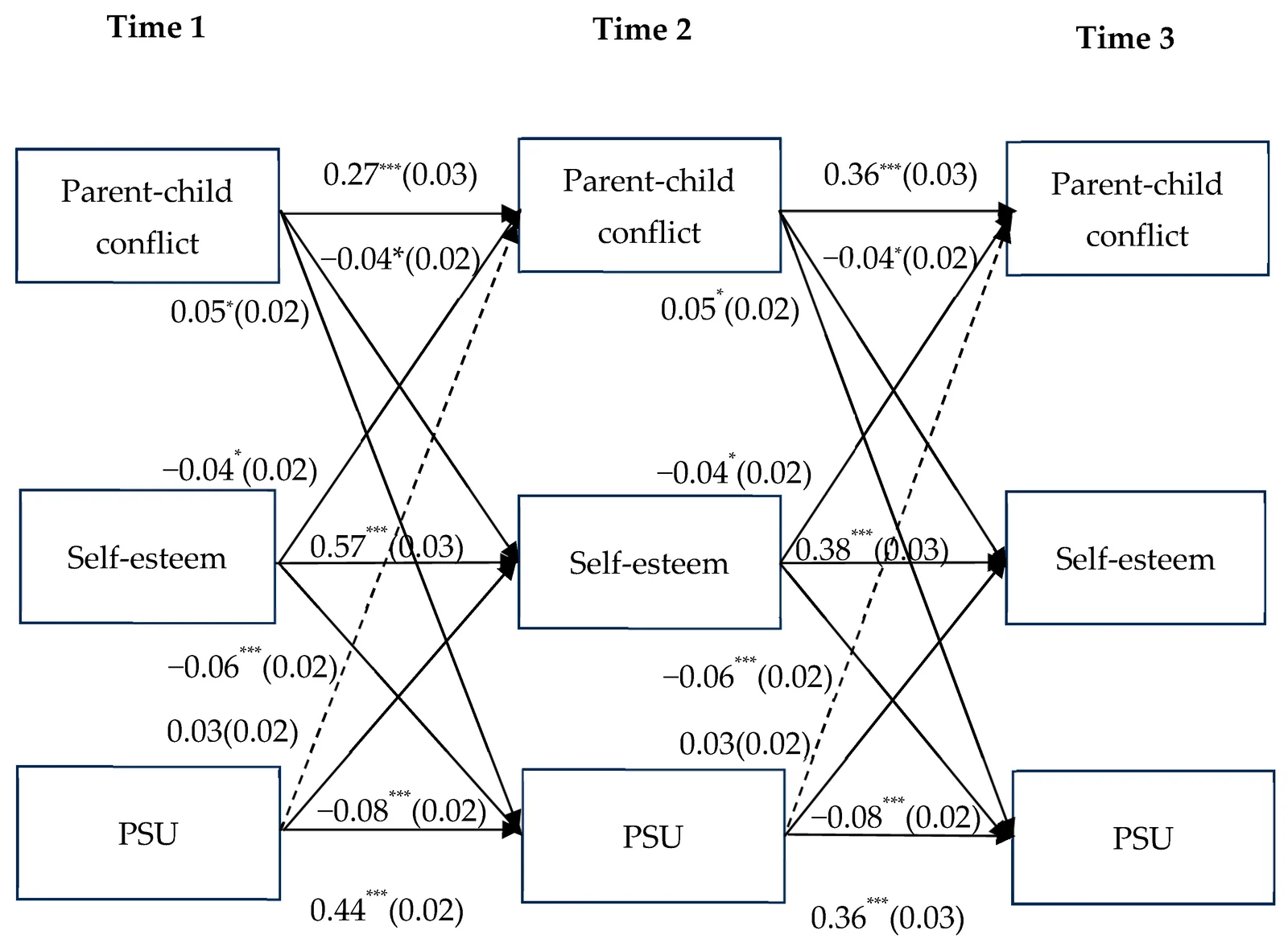
The path coefficients for the CLPM for parent–child conflict, self-esteem, and problematic smartphone use. *Note.* The values outside the parentheses are the standardized path coefficients, and the values inside the parentheses are the standard errors. Dotted lines indicate non-significant relationships. Covariates, within-time correlations and second-order autoregressive paths have been estimated but are not shown in the figure to reduce complexity (* *p* < 0.05, *** *p* < 0.001).

We examined the longitudinal indirect role of self-esteem on the reciprocal association between parent–child conflict and PSU (Figure 2). The mediation model shown good fit to the data: χ^2^(12) = 13.129, χ^2^/df = 1.094, CFI = 1.000, TLI = 0.998, RMSEA = 0.008. As shown in Figure 2, the effects from parent–child conflict to subsequent self-esteem were statistically significant (*β* = −0.04, *SE* = 0.02, *p* < 0.05), and the effects from self-esteem to subsequent PSU were all statistically significant (*β* = −0.06, *SE* = 0.02, *p* < 0.001). Similarly, the effects from PSU to subsequent self-esteem were statistically significant (*β* = −0.08, *SE* = 0.02, *p* < 0.001), and the effects from self-esteem to subsequent parent–child conflict were all statistically significant (*β* = −0.04, *SE* = 0.02, *p* < 0.05). The bootstrap procedure indicated that the path from parent–child conflict to PSU via self-esteem was significant (parent–child conflict→ self-esteem→ PSU; Indirect Effect = 0.002, SE = 0.001, 95%CI [0.001, 0.004]), and vice versa (PSU→ self-esteem→ parent–child conflict; Indirect Effect = 0.003, SE = 0.002, 95%CI [0.001, 0.006]).

### 3.4. Sensitivity Analyses with Family Socioeconomic Covariates

When father’s education, mother’s education, and family SES were additionally controlled, the model fitted the data well, χ^2^(4) = 7.017, χ^2^/df = 1.754, RMSEA = 0.023, CFI = 0.998, TLI = 0.980, SRMR = 0.007. The cross-lagged path from parent–child conflict to PSU remained statistically significant, β = 0.05, SE = 0.02, *p* < 0.01, and the reverse path from PSU to parent–child conflict also remained significant, β= 0.04, SE= 0.02, *p* < 0.05. None of the family-level socioeconomic covariates significantly predicted T2 or T3 parent–child conflict or PSU (all *p* > 0.05). These results suggest that the observed associations were robust to the inclusion of family-level socioeconomic covariates.

### 3.5. RI-CLPM Sensitivity Analysis: Parent–Child Conflict and Problematic Smartphone Use

**Figure 3:**
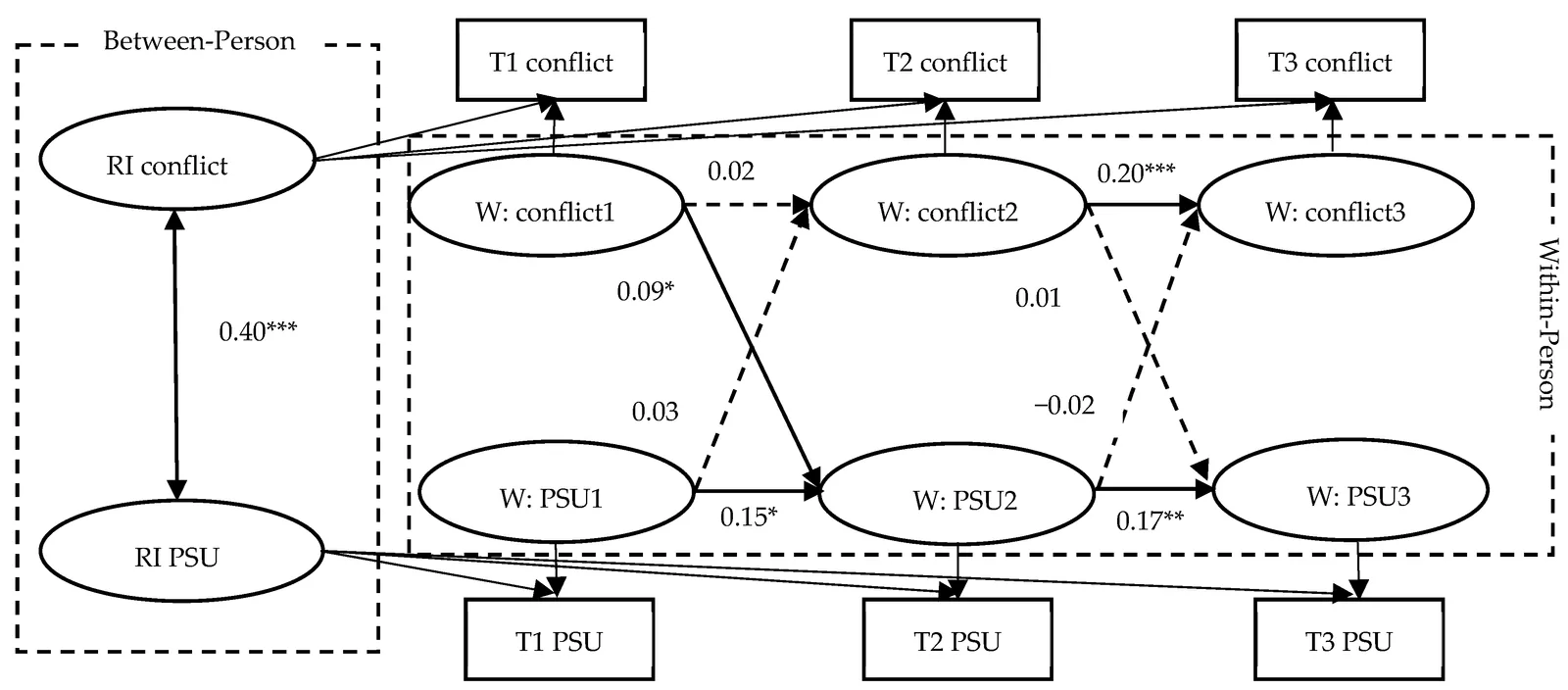
The RI-CLPM model testing the associations between parent–child conflict and problematic smartphone use over time. *Note.* Dotted lines represent nonsignificant paths. The model also includes age and gender as covariates, which are not shown for clarity of presentation. Conflict = parent–child conflict, PSU = problematic smartphone use. * *p* < 0.05; ** *p* < 0.01; *** *p* < 0.001.

The two-variable RI-CLPM fitted the data well, χ^2^(9) = 7.929, RMSEA = 0.000, CFI = 1.000, TLI = 1.000, SRMR = 0.010. At the between-person level, the random intercepts of parent–child conflict and PSU were significantly positively correlated (*β*= 0.40, *p* < 0.001). At the within-person level, the cross-lagged path from T1 parent–child conflict to T2 PSU was significant (β = 0.09, *p* < 0.05), whereas the reverse path from T1 PSU to T2 parent–child conflict was not significant (β = 0.03, *p* > 0.05). The T2 → T3 cross-lagged paths were also non-significant (T2 parent–child conflict → T3 PSU: β = 0.01, *p* > 0.05; T2 PSU → T3 parent–child conflict: β = −0.02, *p* > 0.05). Within-person autoregressive paths were significant for PSU from T1 to T2 (β = 0.15, *p* < 0.05) and T2 to T3 (β = 0.17, *p* < 0.01), and for parent–child conflict from T2 to T3 (β = 0.20, *p* < 0.001), but not from T1 to T2 (β = 0.02, *p* > 0.05). The RI-CLPM for parent–child conflict and PSU is presented in Figure 3.

### 3.6. RI-CLPM Sensitivity Analysis: The Indirect Effect of Self-Esteem on the Association Between Parent–Child Conflict and Problematic Smartphone Use

The three-variable RI-CLPM also fitted the data well, χ^2^(15) = 12.327, RMSEA = 0.000, CFI = 1.000, TLI = 1.003, SRMR = 0.009. At the between-person level, parent–child conflict was negatively correlated with self-esteem (β= −0.20, *p* < 0.01), self-esteem was negatively correlated with PSU (β= −0.49, *p* < 0.001), and parent–child conflict was positively correlated with PSU (β= 0.40, *p* < 0.001). At the within-person level, the only significant cross-lagged path was T1 parent–child conflict → T2 PSU (β = 0.09, *p* < 0.05). The reverse path from T1 PSU to T2 parent–child conflict was not significant (β = 0.03, *p* > 0.05), and the T2 → T3 cross-lagged paths were also non-significant (T2 conflict → T3 PSU: β = 0.01, *p* > 0.05; T2 PSU → T3 conflict: β = −0.02, *p* > 0.05). The within-person indirect effects involving self-esteem were all non-significant (e.g., T1 conflict → T2 self-esteem → T3 PSU: β = −0.001, *p* > 0.05; T1 PSU → T2 self-esteem → T3 conflict: β = 0.000, *p* > 0.05; all 95% CIs included zero). Table 3 presents the detailed results of the RI-CLPM.

## 4. Discussion

Using a three-wave longitudinal design spanning one year, this study examined the longitudinal associations among parent–child conflict, self-esteem, and PSU in Chinese adolescents. Although previous studies have indicated an association between parent–child conflict and PSU, their reliance on cross-sectional designs made it impossible to determine the directionality of these effects. This study addresses several important gaps in the literature. First, by employing traditional CLPMs with three waves of data, it moves beyond correlational evidence to test the temporal ordering and bidirectional nature of the parent–child conflict–PSU association. Second, by applying the I-PACE model, it examines self-esteem as a theoretically grounded mediating mechanism in both directions, rather than treating it as a simple correlate. Third, by supplementing traditional CLPM with RI-CLPM, it distinguishes between-person and within-person processes, revealing that the longitudinal link may partly reflect stable individual differences. Traditional CLPM showed bidirectional predictions between parent–child conflict and PSU, with self-esteem significantly mediating both indirect pathways: parent–child conflict → self-esteem → PSU and PSU → self-esteem → parent–child conflict. RI-CLPM sensitivity analysis revealed significant between-person associations among parent–child conflict, self-esteem, and PSU. At the within-person level, only T1 parent–child conflict prospectively predicted T2 PSU; the reverse path and within-person mediation were not significant. Therefore, the reciprocal pattern should be interpreted mainly as a between-person/traditional CLPM-level correlational pattern rather than as strong evidence for a within-person reciprocal process.

### 4.1. The Longitudinal Association Between Parent–Child Conflict and Problematic Smartphone Use

Consistent with Hypothesis 1, traditional CLPM revealed a significant reciprocal relationship between parent–child conflict and PSU. Specifically, parent–child conflict significantly predicted subsequent PSU, and PSU also significantly predicted subsequent parent–child conflict. In the traditional CLPM, these findings indicated that the association between parent–child conflict and PSU is not unidirectional but rather reciprocal over time. However, the RI-CLPM did not support a fully reciprocal within-person process; the within-person evidence was limited to a prospective path from T1 parent–child conflict to T2 PSU, whereas the reverse path and within-person mediation were not significant. Therefore, the reciprocal pattern observed here should be understood primarily as a between-person or traditional CLPM-level correlational pattern rather than as definitive evidence for a within-person reciprocal mechanism. In line with the family systems theory (Bowen, 1966), the family functions as an integrated emotional system in which all members are interdependent. When adolescents experience persistent conflict with their parents, they may feel emotionally unsupported and unable to cope with family stress. In response, they may withdraw from family interactions and turn to smartphones as a means of escape, comfort, or compensation. This process is consistent with previous findings that parent–child conflict is a risk factor for PSU (Ko et al., 2015; Özaslan et al., 2022; Wei et al., 2025). Thus, the path from parent–child conflict to PSU can be understood as a maladaptive coping process within a distressed family system. Over time, excessive smartphone use may be associated with fewer opportunities for positive family interaction, which may in turn maintain or exacerbate the conflict. When adolescents engage in excessive smartphone use, they may become less available for family interaction, neglect household responsibilities, and respond to parental concerns with irritability or withdrawal. These behaviors can disrupt the emotional balance of the family system and trigger parental reactions such as criticism, control, or heightened monitoring. Such parental responses may, in turn, escalate into more frequent and intense conflict. The present traditional CLPM findings therefore extend previous cross-sectional research by demonstrating a reciprocal correlational pattern between parent–child conflict and PSU at the between-person level, while the RI-CLPM results suggest caution in interpreting this pattern as a within-person reciprocal process. These findings underscore the importance of considering family-level processes in understanding adolescent PSU.

### 4.2. The Longitudinal Indirect Effect of Self-Esteem

Consistent with Hypothesis 2, traditional CLPM results indicated that self-esteem mediated the bidirectional association between parent–child conflict and PSU. In the traditional CLPM, for the path from conflict to PSU, higher parent–child conflict predicted lower self-esteem, which in turn predicted higher PSU. One possible interpretation, consistent with the I-PACE model, is that conflict undermines adolescents’ self-worth and fosters negative self-schemas, which may motivate them to seek temporary relief through smartphone use; this avoidance may become negatively reinforced and contribute to PSU (Brand et al., 2019; X. Wang et al., 2024). This pathway aligns with prior research showing that low self-esteem is a risk factor for problematic mobile phone use (Yang et al., 2024). In the traditional CLPM, for the reverse path, higher PSU predicted lower self-esteem, which in turn predicted greater parent–child conflict. This pattern is consistent with the reverse pathway in the I-PACE model, in which maladaptive behavior feeds back onto personal traits and the environment. One possible interpretation is that excessive smartphone use may deplete psychological resources and interfere with real-world coping and social skills, which may reinforce negative self-schemas and elicit more negative parental reactions, thereby contributing to parent–child conflict (Brand et al., 2019; Chiu, 2014; Elhai et al., 2017). Consistent with this interpretation, Zhao et al. (2026) supported the view that family relational processes are closely intertwined with adolescents’ problematic digital behaviors. However, the RI-CLPM did not support significant within-person mediation in either direction. Therefore, these mediated pathways should be interpreted as traditional CLPM-level indirect correlational patterns rather than as strong evidence for a within-person reciprocal mediation process. This distinction is important because the RI-CLPM provided more limited evidence at the within-person level, with only T1 parent–child conflict prospectively predicting T2 PSU and no significant reverse or within-person mediation path.

We noticed that although the indirect effects of self-esteem were statistically significant, they were relatively small. In longitudinal autoregressive models, effect sizes are often attenuated because stability paths absorb substantial variance (Orth et al., 2024). As Adachi and Willoughby (2015) demonstrated, effect sizes in longitudinal autoregressive models are systematically attenuated because these models control for stability effects on the outcome. Consequently, longitudinal effect sizes that fall below the universal guidelines for ’small‘ may be incorrectly dismissed as trivial, when they might be meaningful.Given that indirect effects are products of path coefficients and are by definition smaller than the direct effects comprising them (Preacher & Hayes, 2008), the observed indirect effect magnitudes are consistent with meaningful, non-trivial pathways in longitudinal mediation models.

### 4.3. RI-CLPM Sensitivity Analysis: Between-Person Associations and Limited Within-Person Reciprocity

We conducted a sensitivity analysis using RI-CLPM. The RI-CLPMs provided a more conservative picture of the longitudinal associations. At the between-person level, parent–child conflict was positively associated with PSU and negatively associated with self-esteem, while self-esteem was negatively associated with PSU. These findings indicate that adolescents with generally higher levels of parent–child conflict also tended to have generally higher PSU and lower self-esteem. At the within-person level, only the path from T1 parent–child conflict to T2 PSU was significant. The reverse within-person path from PSU to later parent–child conflict was not significant, nor were the T2 → T3 cross-lagged paths. In addition, the within-person indirect effects involving self-esteem were all non-significant. These findings suggest that the bidirectional associations observed in the traditional CLPM may partly reflect stable between-person differences rather than clear within-person reciprocal processes. Therefore, the present results should be interpreted as evidence for a stable between-person association among parent–child conflict, self-esteem, and PSU, and a modest within-person prospective link from parent–child conflict to PSU in the first interval, rather than as strong evidence of a dynamic bidirectional developmental process or a within-person mediation through self-esteem.

## 5. Implications and Limitations

These findings suggest that family-based prevention should target parent–child conflict by promoting constructive communication, emotional support, and autonomy-respectful monitoring. Interventions may be more effective if they simultaneously address family relational quality and adolescents’ self-evaluation, although the small indirect effects caution against treating self-esteem as a primary intervention target. Because RI-CLPM indicated that the association largely reflected stable between-person differences, with only T1 conflict → T2 PSU significant at the within-person level, implications should center on parent–child conflict as a correlate of PSU. Strong claims about within-person reverse effects or within-person mediation by self-esteem are not supported.

Limitations include the Chinese adolescent sample, which limits cross-cultural generalizability; self-report measures, which risk common method bias and may not align with parent or objective reports; untested longitudinal measurement invariance; and the inability of traditional CLPM to separate between- and within-person variance, although RI-CLPM showed weak within-person effects. Unmeasured confounders (e.g., adolescent emotional difficulties, parental monitoring, parental smartphone use, family functioning) preclude causal conclusions. The small cross-lagged and indirect effects, together with only four schools and unreliable cluster-robust estimation, warrant caution. Future research should use multi-informant and multi-method designs, test measurement invariance, include more waves and schools, apply multilevel or continuous-time models, and replicate findings with effect sizes in independent samples.

## 6. Conclusions

Guided by the I-PACE model, this study used CLPMs and RI-CLPMs to examine the relationships among parent–child conflict, self-esteem, and PSU. CLPMs showed that parent–child conflict predicted later PSU, and vice versa. Self-esteem mediated the indirect reciprocal relationship between parent–child conflict and PSU. The RI-CLPMs showed a significant between-person association among parent–child conflict, self-esteem, and PSU. At the within-person level, parent–child conflict at T1 prospectively predicted PSU at T2, whereas the reverse within-person path and the within-person mediation through self-esteem were not significant. These findings suggest that the longitudinal link between parent–child conflict and PSU may partly reflect stable between-person differences, with only limited within-person prospective evidence for a conflict-to-PSU pathway.

## Figures and Tables

**Table 1:** Descriptive statistics and correlations for all variables (N = 1368).

	1	2	3	4	5	6	7	8	9	10	11
1. gender	-										
2. age	−0.00	-									
3. T1Parent-child conflict	0.05	0.01	-								
4. T2Parent-child conflict	−0.01	−0.01	0.28 **	-							
5. T3Parent-child conflict	−0.01	0.01	0.27 **	0.41 **	-						
6. T1self-esteem	−0.02	−0.05	−0.10 **	−0.10 **	−0.07 *	-					
7. T2self-esteem	−0.01	−0.02	−0.12 **	−0.07 **	−0.07 *	0.59 **	-				
8. T3self-esteem	0.01	−0.03	−0.09 **	−0.10 **	−0.08 **	0.49 **	0.55 **	-			
9. T1PSU	0.07	0.03	0.30 **	0.15 **	0.14 **	−0.28 **	−0.22 **	−0.22 **	-		
10. T2PSU	0.05	0.02	0.21 **	0.25 **	0.14 **	−0.19 **	−0.24 **	−0.24 **	0.48 **	-	
11. T3PSU	0.02	0.03	0.13 **	0.14 **	0.26 **	−0.19 **	−0.20 **	−0.27 **	0.38 **	0.47 **	-
*M*	0.40	16.05	1.80	1.63	1.49	2.65	2.63	2.72	2.37	2.26	2.20
*SD*	0.49	0.85	0.86	0.80	0.74	0.41	0.38	0.43	0.79	0.80	0.81

*Note.* PSU = problematic smartphone use; * *p* < 0.05, ** *p* < 0.01.

**Table 2:** Fit indices for competing models (N = 1368).

Model	*χ* ^2^	*df*	*RMSEA*	*CFI*	*TLI*	*SRMR*	Δ*χ^2^*	*p*
Model 1	7.433	4	0.025	0.997	0.985	0.009	-	
Model 2	12.035	5	0.032	0.995	0.976	0.016	4.602	0.032
Model 3	14.910	5	0.038	0.992	0.966	0.015	7.477	0.006
Model 4	20.201	6	0.042	0.989	0.960	0.022	12.768	0.002

*Note.* Model 1: full reciprocal bidirectional model; Model 2: unidirectional model retaining only parent–child conflict predicting PSU; Model 3: unidirectional model retaining only PSU predicting parent–child conflict; Model 4: nested model.

**Table 3:** Results of the RI-CLPM for parent–child conflict, self-esteem, and PSU.

Level	Path/Association	β	SE	*p*
Between-person	RI_X ↔ RI_Y (conflict ↔ self-esteem)	−0.20	0.07	0.006
	RI_X ↔ RI_Z (conflict ↔ PSU)	0.40	0.07	<0.001
	RI_Y ↔ RI_Z (self-esteem ↔ PSU)	−0.49	0.06	<0.001
Within-person: autoregressive	T1 conflict → T2 conflict	0.02	0.05	0.766
	T2 conflict → T3 conflict	0.20	0.05	<0.001
	T1 self-esteem → T2 self-esteem	0.23	0.07	0.001
	T2 self-esteem → T3 self-esteem	0.14	0.07	0.058
	T1 PSU → T2 PSU	0.15	0.06	0.015
	T2 PSU → T3 PSU	0.17	0.06	0.004
Within-person: cross-lagged	T1 conflict → T2 self-esteem	−0.06	0.04	0.142
	T2 conflict → T3 self-esteem	−0.04	0.04	0.389
	T1 self-esteem → T2 conflict	−0.04	0.04	0.428
	T2 self-esteem → T3 conflict	0.001	0.04	0.978
	T1 conflict → T2 PSU	0.09	0.04	0.029
	T2 conflict → T3 PSU	0.01	0.04	0.91
	T1 PSU → T2 conflict	0.03	0.05	0.621
	T2 PSU → T3 conflict	−0.02	0.04	0.615
	T1 self-esteem → T2 PSU	0.04	0.05	0.381
	T2 self-esteem → T3 PSU	0.01	0.05	0.754
	T1 PSU → T2 self-esteem	0.01	0.05	0.769
	T2 PSU → T3 self-esteem	−0.05	0.05	0.269

Note. RI_X = random intercept for parent–child conflict; RI_Y = random intercept for self-esteem; RI_Z = random intercept for PSU. Conflict = parent–child conflict; PSU = problematic smartphone use.

## Data Availability

The datasets generated during the present study are not publicly available due to adolescent privacy and ethical restrictions, but de-identified data are available from the corresponding author upon reasonable request.

## References

[B1-behavsci-16-01881] Adachi P., Willoughby T. (2015). Interpreting effect sizes when controlling for stability effects in longitudinal autoregressive models: Implications for psychological science. European Journal of Developmental Psychology.

[B2-behavsci-16-01881] Billieux J., Schimmenti A., Khazaal Y., Maurage P., Heeren A. (2015). Are we overpathologizing everyday life? A tenable blueprint for behavioral addiction research. Journal of Behavioral Addictions.

[B3-behavsci-16-01881] Bowen M. (1966). The use of family theory in clinical practice. Comprehensive Psychiatry.

[B4-behavsci-16-01881] Bradford K., Vaughn L. B., Barber B. K. (2008). When there is conflict: Interparental conflict, parent-child conflict, and youth problem behaviors. Journal of Family Issues.

[B5-behavsci-16-01881] Brailovskaia J., Margraf J. (2020). How to measure self-esteem with one item? Validation of the German single-item self-esteem scale (G-SISE). Current Psychology.

[B6-behavsci-16-01881] Brand M., Wegmann E., Stark R., Müller A., Wölfling K., Robbins T., Potenza M. N. (2019). The Interaction of Person-Affect-Cognition-Execution (I-PACE) model for addictive behaviors: Update, generalization to addictive behaviors beyond internet-use disorders, and specification of the process character of addictive behaviors. Neuroscience & Biobehavioral Reviews.

[B7-behavsci-16-01881] Brand M., Young K. S., Laier C., Wölfling K., Potenza M. N. (2016). Integrating psychological and neurobiological considerations regarding the development and maintenance of specific Internet-use disorders: An Interaction of Person-Affect-Cognition-Execution (I-PACE) model. Neuroscience & Biobehavioral Reviews.

[B8-behavsci-16-01881] Casale S., Fioravanti G., Benucci S. B., Falone A., Ricca V., Rotella F. (2022). A meta-analysis on the association between self-esteem and problematic smartphone use. Computers in Human Behavior.

[B9-behavsci-16-01881] China Internet Network Information Center (2023). The 5th national survey report on internet use of minors in China.

[B10-behavsci-16-01881] Chiu S. I. (2014). The relationship between life stress and smartphone addiction on Taiwanese university student: A mediation model of learning self-efficacy and social self-efficacy. Computers in Human Behavior.

[B11-behavsci-16-01881] Christou A. I., Karampini Z., Kourkoutas E., Bacopoulou F. (2026). Perceived paternal acceptance–rejection, self-perception, and peer victimization in preadolescents with and without special educational needs. International Journal of Environmental Research and Public Health.

[B12-behavsci-16-01881] Crocker J., Wolfe C. T. (2001). Contingencies of self-worth. Psychological Review.

[B13-behavsci-16-01881] Csibi S., Griffiths M. D., Demetrovics Z., Szabo A. (2021). Analysis of problematic smartphone use across different age groups within the ‘components model of addiction’. International Journal of Mental Health and Addiction.

[B14-behavsci-16-01881] Deng L., Fang Y., Wu M., Zhang J., Liu Q. (2013). Family environment, parent-child attachment and adolescent internet addiction. Psychological Development and Education.

[B15-behavsci-16-01881] Efrati Y., Rosenberg H., Ophir Y. (2024). Effective parental strategies against problematic smartphone use among adolescents: A 6-month prospective study. Addictive Behaviors.

[B16-behavsci-16-01881] Elhai J. D., Dvorak R. D., Levine J. C., Hall B. J. (2017). Problematic smartphone use: A conceptual overview and systematic review of relations with anxiety and depression psychopathology. Journal of Affective Disorders.

[B17-behavsci-16-01881] Fang X., Dong Q. (1998). Parent-child conflicts during early adolescence. Psychological Science.

[B18-behavsci-16-01881] Fischer-Grote L., Kothgassner O. D., Felnhofer A. (2021). The impact of problematic smartphone use on children’s and adolescents’ quality of life: A systematic review. Acta Paediatrica.

[B19-behavsci-16-01881] Guo Z. (2023). Relationship between migrant children’s parenting style and self-control: The chain mediation role of family function and self-esteem. Psychological Development and Education.

[B20-behavsci-16-01881] Hu S., Liang L., Yuan K., Bian Y. (2019). The influences of conflicts with parents and friends on loneliness in middle school and its gender difference: A cross-lagged analysis. Psychological Development and Education.

[B21-behavsci-16-01881] Jeong H., Yim H. W., Lee S. Y., Lee J. H. (2020). A partial mediation effect of father-child attachment and self-esteem between parental marital conflict and subsequent features of internet gaming disorder in children: A 12-month follow-up study. BMC Public Health.

[B22-behavsci-16-01881] Jia J. C., Li D. P., Liu Y. X., Zhou Y. Y., Sun W. Q., Wang Y. H., Tong W. (2024). Longitudinal contributions of parent-child and teacher-student relationships to adolescent problematic internet use: The mediating role of self-esteem. Children and Youth Services Review.

[B23-behavsci-16-01881] Jiang X., Lin Y., Liu Q. (2022). The association between parental psychological control and adolescent smartphone addiction: The role of online psychological needs satisfaction and environmental sensitivity. Psychological Development and Education.

[B24-behavsci-16-01881] Kim J. H. (2022). Parental support and problematic smartphone use: A serial mediating model of self-esteem and fear of missing out. International Journal of Environmental Research and Public Health.

[B25-behavsci-16-01881] Kline R. B. (2005). Principles and practice of structural equation modeling.

[B26-behavsci-16-01881] Ko C. H., Wang P. W., Liu T. L., Yen C. F., Chen C. S., Yen J. Y. (2015). Family factors and internet addiction. Psychiatry and Clinical Neurosciences.

[B27-behavsci-16-01881] Kong F., Qin J., Huang B., Zhang H., Lei L. (2020). The effect of social anxiety on mobile phone addiction among Chinese adolescents: A moderated mediation model. Children and Youth Services Review.

[B28-behavsci-16-01881] Leung L. (2008). Linking psychological attributes to addiction and improper use of the mobile phone among adolescents in Hong Kong. Journal of Children and Media.

[B29-behavsci-16-01881] Liang Q., Yu C., Xing Q. (2024). Parent-adolescent conflict, peer victimization, and internet gaming disorder among Chinese adolescents: The moderating effect of OXTR gene rs53576 polymorphism. Child Psychiatry & Human Development.

[B30-behavsci-16-01881] Lin S., Li Y., Sheng J., Chen L., Zhang Y., Chen J. (2025). Non-suicidal self-injury in Chinese adolescents with problematic smartphone use: A one-year longitudinal study. Children and Youth Services Review.

[B31-behavsci-16-01881] Liu Q. Q., Zhang D. J., Yang X. J., Zhang C. Y., Fan C. Y., Zhou Z. K. (2018). Perceived stress and mobile phone addiction in Chinese adolescents: A moderated mediation model. Computers in Human Behavior.

[B32-behavsci-16-01881] Liu Y., Shi Y., Zhang L., Wang Y. (2025). Rumination mediates the relationships between social anxiety and depression with problematic smartphone use in Chinese youth: A longitudinal approach. International Journal of Mental Health and Addiction.

[B33-behavsci-16-01881] Mathew P., Krishnan R. (2020). Impact of problematic internet use on the self-esteem of adolescents in the selected school, Kerala, India. Archives of Psychiatric Nursing.

[B34-behavsci-16-01881] Orth U., Meier L. L., Bühler J. L., Dapp L. C., Krauss S., Messerli D., Robins R. W. (2024). Effect size guidelines for cross-lagged effects. Psychological Methods.

[B35-behavsci-16-01881] Orth U., Robins R. W. (2013). Understanding the link between low self-esteem and depression. Current Directions in Psychological Science.

[B36-behavsci-16-01881] Özaslan A., Yıldırım M., Güney E., Öztürk M. (2022). Association between problematic internet use, quality of parent-adolescents relationship, conflicts, and mental health problems. International Journal of Mental Health and Addiction.

[B37-behavsci-16-01881] Panova T., Carbonell X. (2018). Is smartphone addiction really an addiction?. Journal of Behavioral Addictions.

[B38-behavsci-16-01881] Peng Y., Cui W., Yang R., Wang H. (2025). Parental attachment and problematic smartphone use in adolescents: The chain-mediated role. Frontiers in Psychology.

[B39-behavsci-16-01881] Preacher K. J., Hayes A. F. (2008). Asymptotic and resampling strategies for assessing and comparing indirect effects in multiple mediator models. Behavior Research Methods.

[B40-behavsci-16-01881] Rosenberg M., Schooler C., Schoenbach C., Rosenberg F. (1995). Global self-esteem and specific self-esteem: Different concepts, different outcomes. American Sociological Review.

[B41-behavsci-16-01881] Snegha J., Sudha M. (2025). Exploring the adverse impact of smartphone use on young individuals’ self-esteem: A structural equation modeling approach based on five temperaments. Engineering, Technology & Applied Science Research.

[B42-behavsci-16-01881] Sohn S. Y., Rees P., Wildridge B., Kalk N. J., Carter B. (2019). Prevalence of problematic smartphone usage and associated mental health outcomes amongst children and young people: A systematic review, meta-analysis and GRADE of the evidence. BMC Psychiatry.

[B43-behavsci-16-01881] Tao S., Wu X., Zhang Y., Zhang S., Tong S., Tao F. (2017). Effects of sleep quality on the association between problematic mobile phone use and mental health symptoms in Chinese college students. International Journal of Environmental Research and Public Health.

[B44-behavsci-16-01881] Tian L. M., Liu L., Nan S. (2018). Parent–child relationships and resilience among Chinese adolescents: The mediating role of self-esteem. Frontiers in Psychology.

[B45-behavsci-16-01881] Van den Eijnden R. J. J. M., Spijkerman R., Vermulst A. A., Engels R. C. M. E. (2010). Compulsive internet use among adolescents: Bidirectional parent–child relationships. Journal of Abnormal Child Psychology.

[B46-behavsci-16-01881] Wang A., Guo S., Chen Z., Li J. (2024). The chain mediating effect of self-respect and self-control on the relationship between parent-child relationship and mobile phone dependence among middle school students. Scientific Reports.

[B47-behavsci-16-01881] Wang X., McGowan A. L., Fosco G. M., Falk E. B., Bassett D. S., Lydon-Staley D. M. (2024). A socioemotional network perspective on momentary experiences of family conflict in young adults. Family Process.

[B48-behavsci-16-01881] Wei W. X., Zhao X. X., Chooi W. T. (2025). Family conflict is associated with increased risk of problematic digital media use in children and adolescents: A systematic review with narrative synthesis. Adolescent Research Review.

[B49-behavsci-16-01881] Yang X., Liu Q., Gao L., Wang G., Liu T. (2024). Linking self-esteem to problematic mobile phone use: A moderated mediation model of fear of missing out and ruminative subtypes. Behavioral Sciences.

[B50-behavsci-16-01881] Zhang J. H., Zhang X. Q., Zhang K. X., Li Y. (2022). An updated meta-analysis on the relationship between mobile phone addiction and sleep disorder. Journal of Affective Disorders.

[B51-behavsci-16-01881] Zhao L., Xiao M., Tang B., Gao Y., Hu D. (2026). How perceived parental technoference relates to adolescent smartphone addiction: Evidence from a moderated mediation model. Behavioral Sciences.

[B52-behavsci-16-01881] Zhou H., Wang S., Lin Y., Jiang H., Lu J. (2024). Harsh parenting and problematic smartphone use: The chain mediating effects of attention to negative information and social anxiety. Journal of Affective Disorders Reports.

[B53-behavsci-16-01881] Zhu Y. L., Deng L. Y., Wan K. (2022). The association between parent-child relationship and problematic internet use among English- and Chinese-language studies: A meta-analysis. Frontiers in Psychology.

